# Directional Effect of Plasticity Ball Burnishing on Surface Finish, Microstructure, Residual Stress and Hardness of Laser Direct Energy Deposited Stellite 21 Alloy

**DOI:** 10.3390/ma18132971

**Published:** 2025-06-23

**Authors:** Mohammad Uddin, Joel Rech, Colin Hall, Thomas Schlaefer

**Affiliations:** 1 UniSA STEM, University of South Australia, Mawson Lakes, SA 5095, Australia; 2 ENISE, Centrale Lyon, 42023 Saint Etienne cedex 2, France; joel.rech@enise.fr; 3 Future Industries Institute, University of South Australia, Mawson Lakes, SA 5095, Australia; colin.hall@unisa.edu.au; 4 Laserbond Ltd., Cavan, SA 5094, Australia; thomass@laserbond.com.au

**Keywords:** ball burnishing, grain modification, laser direct energy deposition, micro-hardness, residual stress, surface integrity, Stellite 21 alloys

## Abstract

This paper investigates the effect of plasticity ball burnishing on characteristics of surface integrity, residual stress and hardness of laser direct energy deposited (DEDed) Stellite 21 alloys, with a focus on the burnishing directional effect on surface and microstructural deformation. The results demonstrated that the burnishing improved surface finish, reducing Sa and Sz by 24% and 47%, respectively. The burnishing flattened and modified the cellular/columnar grains at a depth of 50 µm, with the most notable changes observed on the cross-sectional plane normal to the burnishing direction. Compared to the ground surface, the burnishing introduced higher and deeper compressive stresses along normal to the burnishing/grinding direction (−1341 MPa and 61 µm) as compared to that along the burnishing direction (−449 MPa and 56 µm). Likewise, the burnishing increased the full width at half maximum (FWHM) in the same fashion by broadening XRD peaks along normal to the burnishing direction. Due to higher grain modification and dislocation density, the burnishing has improved microhardness at a depth of 320 µm by 26% along normal to the burnishing direction. These findings demonstrate that the plasticity ball burnishing has a directional effect on plastic deformation and can be considered a plausible technique for tailored surface integrity, residual stress and hardness, which potentially improve the service performance of DEDed Stellite 21 alloy components.

## 1. Introduction

Stellite 21, a Co-based hard-facing alloy, is widely used in high-wear applications, such as the impellers of abrasive slurry pumps, brake pads, and rail flange-ramp contacting components [1]. Laser metal direct energy deposition (DED) has become a common technique for depositing hard-facing alloy coatings to repair or restore industrial components [2]. In DED, metal powders are melted by a laser and rapidly cooled, forming deposited layers in an upward build direction. This intrinsic directional heating and cooling result in an inhomogeneous microstructure, high surface roughness, and tensile surface stress, which can negatively impact functional performance, including fatigue resistance, corrosion, and wear life [3].

Ganesh et al. (2010) [4] observed a deterioration in fracture resistance due to crack propagation under tensile loading in laser-clad Stellite 21, a phenomenon also reported for Stellite 6 alloys in [5]. This indicates that laser-clad Stellite surfaces often require post-treatment to improve surface integrity and functional properties. Mechanical surfacing techniques, such as machining, turning, and grinding, are commonly used to remove rough layers and achieve desired geometric tolerance and surface finish [6,7,8]. Chomienne et al. (2023) [9] highlighted that surface engineering strategies, including turning, rolling, and belt grinding, can be used to tailor surface integrity features, such as low surface roughness and a thick compressive layer, which are critical for the fatigue strength of 15-5 PH steel.

Other techniques, such as shot peening, ultrasonic nano-surface treatment (UNSM), and laser shock peening, have been applied to alter the microstructure, induce compressive residual stress, and increase hardness in surface and subsurface layers [10,11,12]. However, these methods often generate high roughness and cracks due to random impact loading, which can lead to crack initiation and localized tensile stress, despite inducing subsurface grain refinement and compressive stress.

An alternative approach, ball burnishing (BB), is increasingly used to modify laser-deposited DED components. BB plastically deforms the top surface layer by pressing a ball roller against it [13]. BB not only smooths the surface and induces microstructural modification but also reverses tensile stress to compressive stress in the surface/subsurface layers at a deeper level. The efficacy of BB in improving surface integrity and functional performance for DEDed metals has been extensively demonstrated [14,15].

Anirudh et al. (2020) [16] investigated cryogenic-assisted burnishing on laser-clad Stellite 6, reporting improvements in hardness and residual stress profiles. Burnishing has also been shown to transform detrimental tensile stress into beneficial compressive stress in C45 laser alloys by modifying grains up to 30 µm in depth [17]. Courbon et al. (2019) [18] reported similar findings when turning followed by roller burnishing was applied to laser-clad 17-4 PH deposits, improving surface integrity. However, they found less effect of burnishing on cold-sprayed 17-4 PH coatings, suggesting that the level of burnishing effect depends on the intrinsic characteristics of the deposited coating microstructure.

Manji et al. (2024) [19] investigated parallel and cross burnishing path strategies on wire-arc additively manufactured (WAAMed) AZ31 alloy, demonstrating that the cross path resulted in greater depth of grain refinement and surface compressive stress, though the reason for the directional effect was not explained. Similarly, Chomienne et al. (2016) [13] observed variations in residual stress along the axial and circumferential directions of 15-5 PH steel after turning and burnishing. Thit et al. (2025) [20] applied a grinding-burnishing approach on DEDed SS 431 alloy (martensitic steel), demonstrating positive effects on fatigue strength. Liu et al. (2024) [21] introduced in-situ ultrasonic roller burnishing in laser-deposited 316L, resulting in grain transformation and deeper modification, which enhanced corrosion resistance.

Most previous research has focused on surface and residual stress profiles along a specific direction of interest under certain burnishing parameters. However, during burnishing, plastic deformation and material flow occur in three primary directions—longitudinal, transverse, and normal—relative to the burnishing tool’s movement. These directions can influence the degree of change in surface topography, microstructure, and affected depth. The directionality of burnishing is thus a crucial factor, and understanding its impact on deformation mechanisms will help tailor burnishing path strategies to achieve better surface integrity.

In DED, the laser scanning and pitch (overlap) directions also influence the formation and distribution of surface undulations, microstructure, and hardness. However, the effect of burnishing direction on plastic deformation, grain modification, and residual stress in relation to the laser DED directions has not been fully explored in the literature.

Stellite 21 alloys, with their Co-rich matrix, are relatively new in additive manufacturing for harsh applications. Unlike steel alloys, which have favorable work-hardening effects, the plastic deformation mechanisms of Stellite 21 alloys are not well understood. Recently, Sun et al. (2025) [22] applied ultrasonic surface rolling (USR) on laser-cladded Stellite 6 alloys, demonstrating improvements in surface finish, hardness, surface compressive residual stress, and equiaxed grain formation. However, to the best of the authors’ knowledge, there has been little research on the ball burnishing of laser DEDed Stellite 21 alloys and its effects on surface integrity, hardness, and residual stress, particularly in terms of burnishing and DED directionality. Understanding these effects will clarify directional performance and inform the design of surface treatment strategies for tailored service performance.

**Figure 1:**
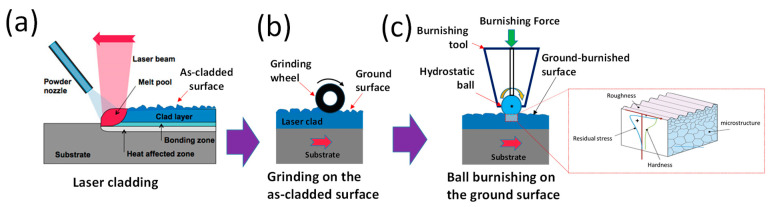
The proposed grinding–burnishing strategy applied on laser metal deposited Stellite 21 alloy: (**a**) laser cladding, (**b**) grinding on cladded surface, and (**c**) burnishing on ground surface.

This study aims to address these gaps. We present a ball burnishing (BB) surface treatment strategy applied to laser DEDed Stellite 21 alloys. Figure 1 illustrates the proposed treatment approach: the as-DEDed surface is first ground to remove the rough layer, followed by plasticity ball burnishing. The goal is to comprehensively investigate the directional effects of this combined treatment on surface roughness, microstructure, residual stress, and microhardness. To explore the directional effect, the treated specimen’s surface was metallurgically sectioned along two planes: (1) along the burnishing direction and (2) normal to the burnishing direction. Extensive characterization using optical profilometry, SEM, XRD, and hardness testing was conducted to analyze the changes in microstructure, surface/profile residual stress, and hardness due to the burnishing directionality.

## 2. Materials and Methods

### 2.1. Direct Metal Deposition and Specimen Preparation

**Figure 2:**
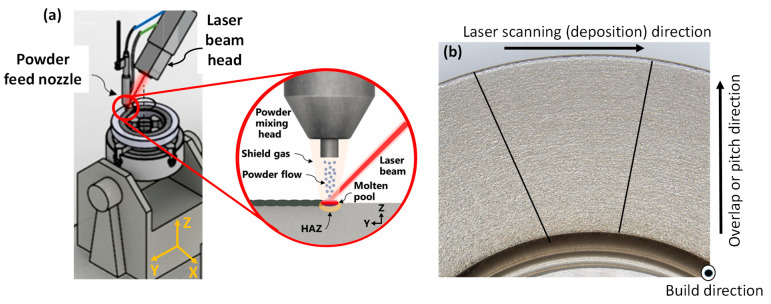
(**a**) Illustration of laser metal deposition of Stellite 21 alloy annular disc, and (**b**) cut section of the deposited block showing laser scanning, overlap or pitch and build directions.

A 1 mm thick Stellite 21 alloy coating was deposited onto an annular disc substrate of mild steel G250 using direct laser energy deposition. The deposition process followed a circular scanning path with an 80% overlap ratio and a 2 mm radial pitch distance, starting from the center of the substrate. An illustration of the laser DED process is provided in Figure 2.

The deposition experiments were conducted in a helium–argon gas flow chamber to prevent oxidation and contamination of the molten metal. The process parameters used during deposition are outlined in Table 1. To prepare the substrate, it was preheated to 200 °C using an oxyfuel torch.

The Stellite 21 powders used were water-atomized, with a mean powder size of approximately 110 µm, and a size range of 63 to 180 µm, as supplied by the manufacturer. The chemical composition of Stellite 21 alloy and the G250 substrate is summarized in Table 2.

The surface topography of the as-clad sample was found to be notably rough, with a Ra value of 29 μm and an Rz value of 124 μm, which is typical of additively manufactured surfaces. To remove the highly rough surface layer, the clad surface was ground using a surface grinding machine (BMT 4080 AH, Hare and Forbes, Melbourne, Australia) with a diamond wheel at 1450 rpm. This was done in five passes, each removing a thin 5 μm layer, for a total removal of 30 μm from the top surface. The grinding process aimed to minimize the introduction of further stress into the laser-clad surface prior to the subsequent burnishing process. The grinding was carried out along the laser cladding (scanning) direction, with cooling lubricant applied during the process. After grinding, the block was cut into coupons measuring 40 mm (L) × 40 mm (W) × 11 mm (H), and the ground surface was referred to as the “Ground” specimen. 

### 2.2. Ball Burnishing Surface Treatment

**Figure 3:**
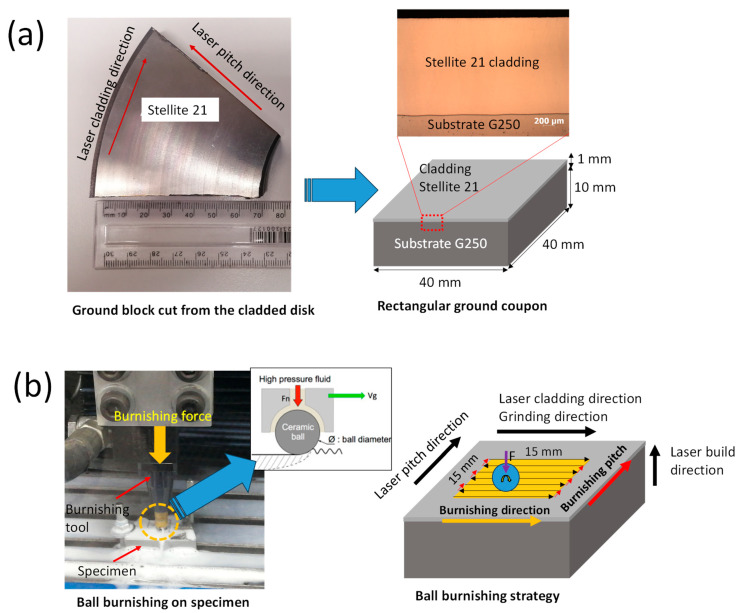
(**a**) Sample preparation (inset photo shows Stellite 21 cladding layer on substrate G250) and (**b**) ball burnishing experimental setup and strategy (inset photo shows the burnishing mechanism).

The ball burnishing (BB) process was performed on a central 15 mm × 15 mm area of a rectangular ground coupon with dimensions of 40 mm × 40 mm × 11 mm (Figure 3a), using an HG6-9 E00° burnishing tool from Ecoroll (Celle, Germany). In this process, a hydraulically pressurized ball deformed the material. The burnishing ball, made of hard SiC, had a diameter of 6 mm. A schematic of the burnishing experimental setup is shown in Figure 3b. The burnishing was carried out at a hydraulic pressure of 160 bar, corresponding to a burnishing force of 452 N. A previous study by the current authors showed that the burnishing force of this magnitude is adequate to induce the plastic deformation, improving surface integrity [14]. The feed rate was set to 500 mm/min, and the stepping distance (pitch) was maintained at 0.1 mm.

As illustrated in Figure 3b, a zigzag continuous burnishing path strategy was employed, with the burnishing direction aligned with either the grinding or laser scanning direction, while the burnishing step-over was perpendicular to the grinding direction. The specimen treated by burnishing is referred to as “Ground + Burnished.” In the subsequent sections, the comparison and discussion of surface integrity results will focus on the differences between the “Ground” and “Ground + Burnished” specimens.

### 2.3. Characterisations

#### 2.3.1. Surface Roughness

Surface roughness was measured using a high-resolution optical confocal microscope (Alicona Infinite Focus by Bruker Alicona, Graz, Austria) equipped with a 20× objective lens. The scanning region of interest (ROI) covered an area of 2.9 mm × 2.9 mm, with a sampling distance of 0.438 µm × 0.438 µm. The system’s cut-off filter, vertical resolution, and lateral resolution were set to 800 µm, <50 nm, and 2.94 µm, respectively, to ensure accurate capture of surface roughness features. Following the scan, form (plane) and surface waviness were filtered out to minimize errors due to surface skewness. Roughness parameters, including 2D profile roughness (Ra, Rz) and 3D surface roughness (Sa, Sz), along with topographical images, were obtained. Profile roughness was measured in directions perpendicular to the grinding line, laser cladding, and burnishing orientations. Measurements were taken at three different locations on each specimen coupon, and the average value was calculated as the final roughness measurement.

#### 2.3.2. Microstructure

**Figure 4:**
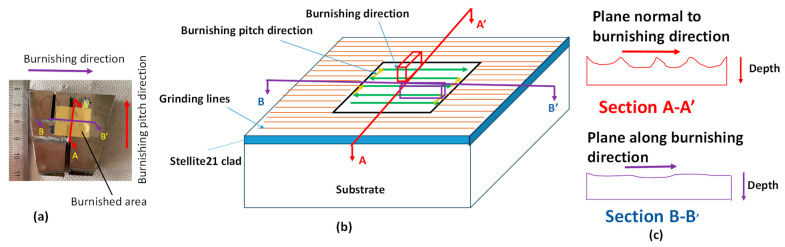
Illustration of sectioning of specimen for microstructural observation (**a**) actual burnished sample, (**b**) cross-sectional planes along the burnishing and pitch directions, and (**c**) the schematic diagram of corresponding sectional surfaces along depth on which microstructural observations were made.

To examine microstructural changes both along the burnishing direction and perpendicular to it, the specimen was cross-sectioned, ground, polished, and electro-etched. Figure 4 illustrates the specimen sectioning protocol for microstructural analysis. The cross-sectional surface along the depth, cut by the B-B’ plane, is referred to as the “burnishing directional plane,” while the cross-section cut by the A-A’ plane is designated as “normal to the burnishing direction” or the “burnishing pitch direction.”

The sectioned surface was ground in stages using SiO_2_ sandpaper: P#240 for 2 min, P#600 for 2 min, and P#1200 with water for 4 min, utilizing the BUEHLER AutoMet 300 machine (BUEHLER, Lake Bluff, IL, USA). Following grinding, the sample was polished on a cloth pad with diamond particles as follows: 9 µm for 2 min, followed by 3 µm for 2 min. This was then succeeded by polishing with alumina colloidal particles of 0.05 µm (pH 9.8) for 4 min to achieve a smooth, shiny, and crack-free surface. The sample was then electro-etched using a 4 wt% oxalic acid electrolyte at 5 V for 5 s to reveal the microstructure. Microstructural topography was captured using both Bruker’s TESCAN scanning electron microscope (TESCAN, Brno, Czech Republic) and Zeiss’s AX10 HAL 100 optical microscope (Zeiss, Oberkochen, Germany).

#### 2.3.3. XRD

**Figure 5:**
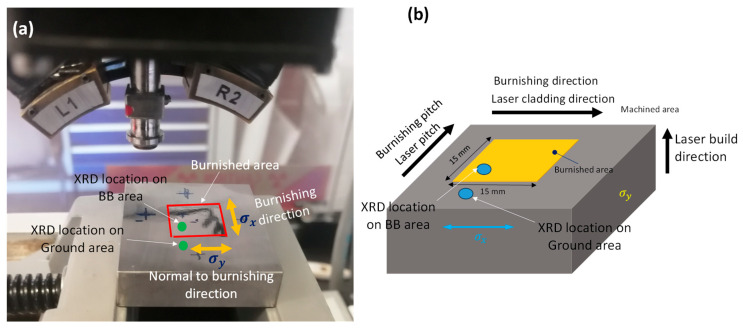
(**a**) XRD measurement setup, and (**b**) illustration of the locations for residual stress measurement along the depth.

Residual stress was measured on the ground and burnished specimens using Proto’s iXRD system. Figure 5 shows an XRD experimental setup indicating the location of measurement on the sample. Residual stress measurements were conducted on both burnishing ($\sigma_{x}$) and normal to burnishing ($\sigma_{y}$) directions. Table 3 summarises residual stress measurement parameters used in this study.

The in-depth residual stresses were measured after layer-by-layer material removal using Proto’s electrolytic polisher (at 45 V and 3.5 A) in an ammonium chloride solution. After each layer removal, the depth was measured using a Talysurf profilometer (from Taylor Hobson, Leicester, UK). The amount of depth to be removed was determined by the time in electropolishing. Sin^2^ Ψ vs. d-spacing method was used to estimate the residual stress. Peak breadths, often termed as FWHMs (full width at half maximum), were extracted from the X-ray diffraction measurements. The diffraction peak breadth of the studied material is mainly related to the grain size and dislocation density.

#### 2.3.4. Microhardness

Microhardness was measured using the Innovatest Falon 600 G2 hardness tester (Innovatest, Maastricht, The Netherlands), which is equipped with a diamond indenter in the shape of a square pyramid with a point angle of 148°. Indentations were made on the polished cross-sectional surfaces along the depth, starting from the top edge, in accordance with the protocol illustrated in Figure 4. The primary objective was to examine how hardness changes in two directions of the sample, influenced by plastic deformation and material flow during the burnishing process.

Indentations were performed along three lines on each surface, with a spacing of 80 µm between consecutive indents along the depth and 220 µm between adjacent lines. This setup produced 31 data points along each depth line, covering approximately 2.48 mm, resulting in a total of 91 indents (31 indents per line × 3 lines). The first indent was positioned 80 µm below the top edge of the surface. For each indentation, the applied load was maintained at F = 0.1 kgf (1 N), with a holding time of 10 s. The average of diagonals of the indents (d_avg_) were measured by the optical microscope, and the hardness was estimated using the formula as shown in Equation (1).

(1) $$\mathrm{HV}0.1=0.189\times \frac{F_{kgf}}{d_{avg}^{2}}$$

## 3. Results

### 3.1. Surface Roughness and Topography

**Figure 6:**
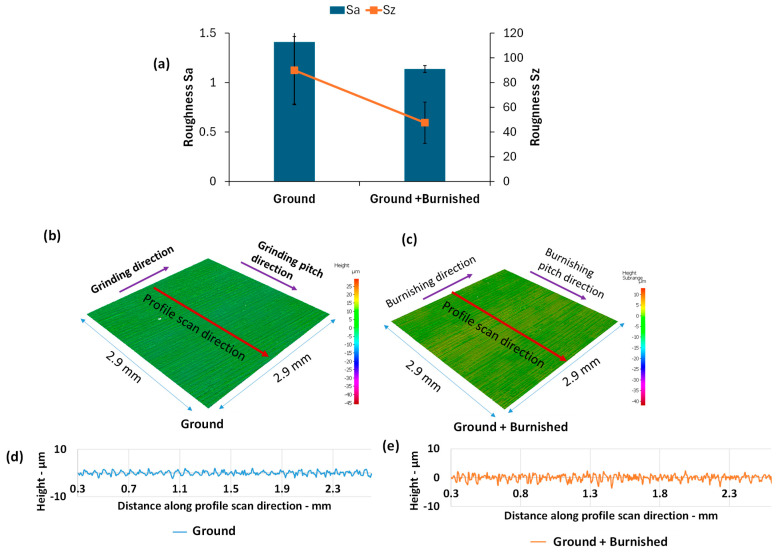
Comparison of surface roughness (**a**) Sa and Sz, (**b**,**c**) 3D surface topography, and (**d**,**e**) roughness profile between Ground and Ground + Burnished specimens.

As demonstrated in Figure 6, the burnishing significantly enhanced the surface roughness of the ground surface. Specifically, compared to the ground surface, the burnishing reduced the S_a_ and S_z_ values by 24% and 47%, respectively (Figure 6a). The plastic deformation induced by burnishing led to the flow of material from the surface peaks into the valleys, resulting in a smoother, more uniform surface topography (Figure 6b,c). This improvement is further evident in the profile height scans taken along a direction perpendicular to the burnishing (Figure 6d,e).

### 3.2. SEM and EDS Analysis

**Figure 7:**
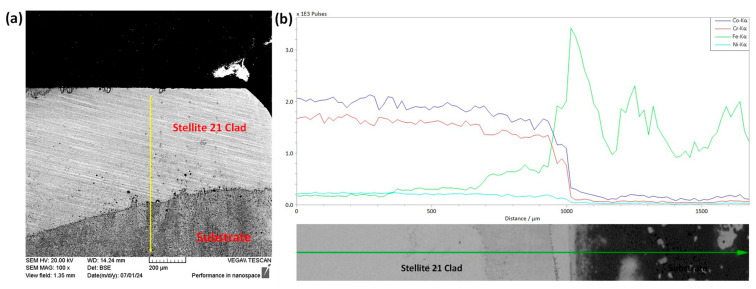
(**a**) Interface of Stellite 21 cladding with G250 substrate and (**b**) their EDS profile showing the major chemical compositions. The yellow line arrow in (**a**) and green line arrow in (**b**) are the EDS scan direction taken during measurement.

SEM and EDS analyses were conducted on the interface between the Stellite 21 coating and the G250 substrate. As shown in Figure 7, a high concentration of Co and Cr elements was observed in the cladded layer, while Fe was predominantly detected in the substrate. These observations further confirm the successful laser direct energy deposition of the Stellite 21 coating with the desired elemental compositions.

**Figure 8:**
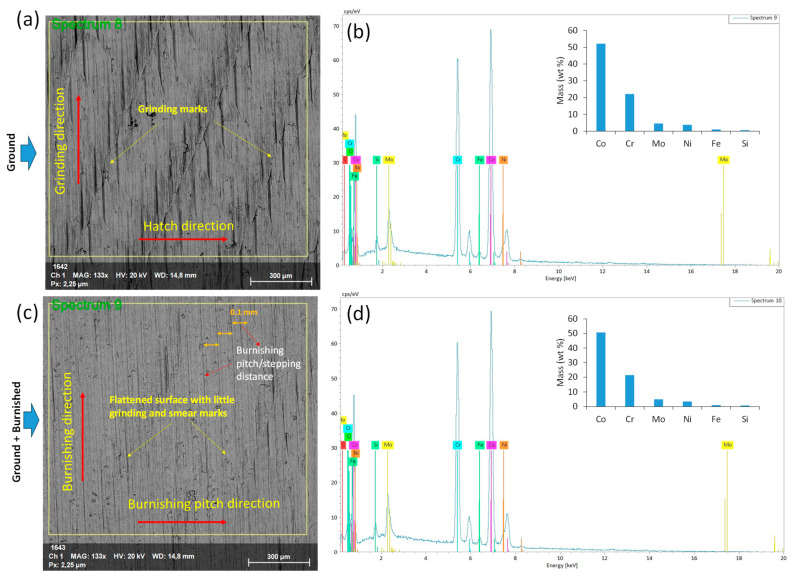
SEM image and EDS profile for (**a**,**b**) ground and (**c**,**d**) ground + burnished surfaces. The yellow squares in (**a**,**c**) define the surface areas over which corresponding EDS spectrums were estimated. Inset bar plots in (**b**,**d**) show a comparison of total mass (wt%) of major relevant elemental compositions.

Figure 8a,c present SEM images of the ground and burnished top surfaces. The ground surface exhibited distinct linear grinding marks and visible surface undulations, which are consistent with the findings of [23]. Additionally, the combination of heat generation and the high-speed rotation of the abrasive wheel in grinding might have caused surface tearing and abrasive adhesion, as evidenced in Figure 8a. In contrast, as can be seen from Figure 8c, the burnishing effectively smoothed the surface, removing the grinding marks but leaving minimal smear traces.

EDS spectra in Figure 8b,d show the corresponding surface chemical compositions. The mass percentages (wt%) of the key elements in the Stellite 21 coating remained unchanged for both the ground and ground + burnished surfaces, indicating that the burnishing did not contaminate the treated surface. The notable elemental composition, including Co (51%), Cr (22%), Ni (5%), and Mo (5%), remained consistent along the depth, as shown in Figure 7.

### 3.3. Microstructural Analysis

**Figure 9:**
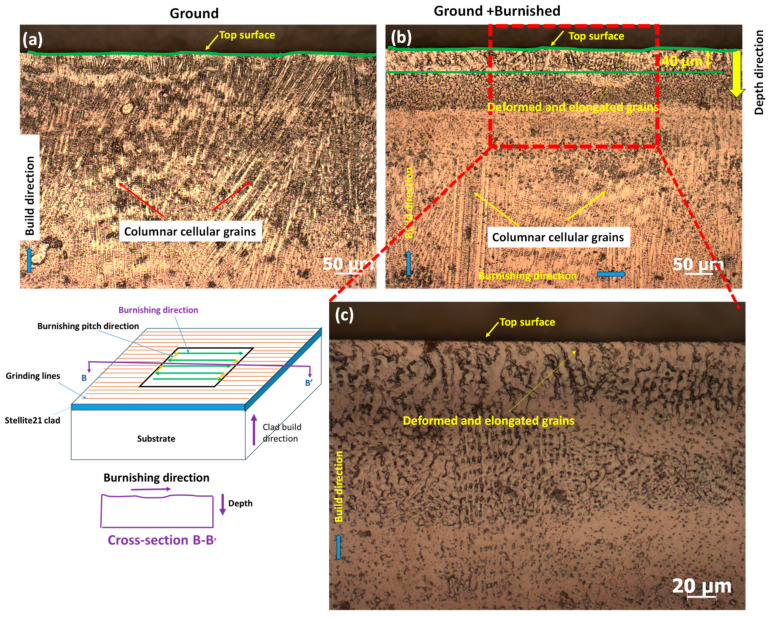
Comparison of microstructure of cross-sectional surface along the burnishing/grinding direction (B-B’ plane) for (**a**) Ground, (**b**) Ground + Burnished surfaces and (**c**) magnified view of top modified layer of the Ground + Burnished surface shown in (**b**).

As illustrated in Figure 9, the microstructures were analyzed to examine the effects of deformation and material flow along the burnishing direction (B-B’ plane). Columnar cellular grains of a Co-rich matrix (white regions) and interdendritic carbide phases (black/gray spots) were observed on both the ground (Figure 9a) and ground + burnished surfaces (Figure 9b). This grain structure is a typical result of the directional rapid heating and cooling mechanisms in the DED process of Stellite 21. No significant change in surface undulation was observed between the ground and burnished surfaces. However, as shown in Figure 9b, compared to the ground surface, the burnishing resulted in the deformation and elongation of grains approximately 40 µm below the top surface. The influence of burnishing on grain straining and shape modification decreased with depth, from the top surface toward the bulk of the material. This effect is further highlighted in the magnified view of the top modified layer on the burnished surface in Figure 9c.

**Figure 10:**
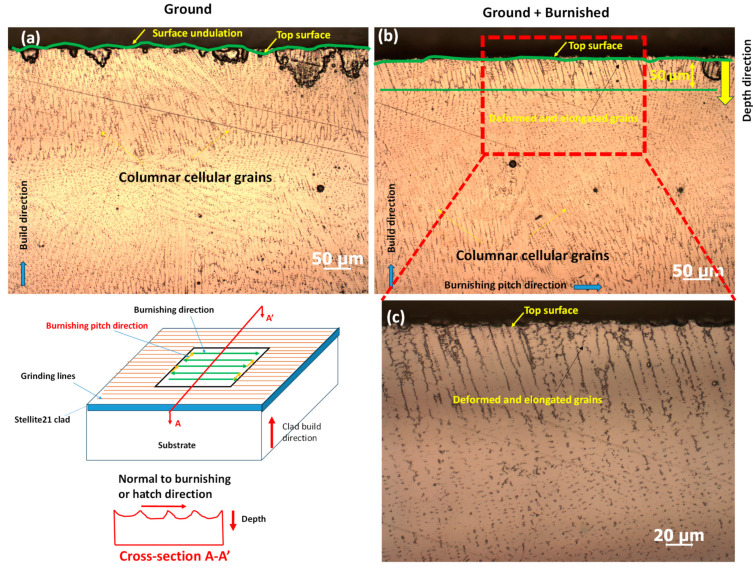
Comparison of microstructure of cross-sectional surface normal to the burnishing/grinding direction (A-A’ plane) for (**a**) Ground, (**b**) Ground + Burnished surfaces, and (**c**) magnified view of top modified layer of the Ground + Burnished surface shown in (**b**).

In Figure 10a, the ground surface, when viewed normal to the grinding direction (A-A’ plane), exhibited undulations or a wavy profile. However, the burnishing process effectively flattened the undulated surface at the top edge, as shown in Figure 10b. Additionally, the burnished surface displayed more deformed and elongated grains extending as deep as 50 µm beneath the top edge, compared to a 40 µm modification layer along the burnishing direction (B-B’ plane) (Figure 9b). This is attributed to the fact that, when burnishing normal to the grinding direction, the peaks of the ground surface experience more plastic deformation, causing material to flow into the valleys and smooth the surface. In contrast, this effect is less pronounced when burnishing along the grinding direction (B-B’ plane).

Therefore, the burnishing induced grain straining and altered grain shapes, which could lead to compressive residual stresses and increased hardness. However, it is important to note that no significant grain refinement due to burnishing was observed in either of the cross-sectional surfaces (A-A’ and B-B’ planes) of the DED-produced Stellite 21 alloys.

### 3.4. Residual Stress

**Figure 11:**
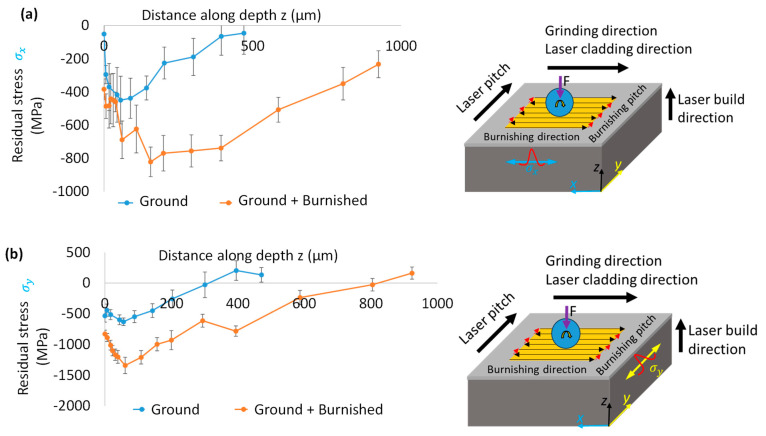
Comparison of residual stress with respect to distance along depth induced by grinding and burnishing along (**a**) the burnishing direction ($\sigma_{x}$) on B-B’ plane and (**b**) normal to the burnishing direction ($\sigma_{y}$) on A-A’ plane. Right side images in (**a**,**b**) depict an illustration of burnishing path and stress measurement directions.

Figure 11 compares the residual stress along the burnishing direction (σ_x_) on the B-B’ plane and perpendicular to the burnishing direction (σ_γ_) on the A-A’ plane. As shown in Figure 11a, the residual stress σ_x_ after grinding and burnishing exhibits compressive behavior from the top surface to the depth. For the ground surface, the maximum compressive stress of −449 MPa occurs at a depth of 56 µm from the top surface, gradually transitioning to nearly tensile stress at a depth of 471 µm. In contrast, after burnishing, the residual stress σ_x_ remains compressive from the top surface, reaching a maximum of −821 MPa at a greater depth of 157 µm. From this point, the magnitude of compressive stress decays, with the compressive stress layer extending as deep as 924 µm. While grinding induced compressive stress, the burnishing produced a significantly higher and deeper compressive stress layer within the cladding.

On the other hand, Figure 11b illustrates the residual stress σ_γ_ (normal to the burnishing direction on the A-A’ plane). After grinding, σ_y_ was compressive, with a maximum of −533 MPa at the surface, peaking at −631 MPa at 56 µm, before becoming tensile at a depth of 300 µm. Following burnishing, σ_γ_ remained compressive, with the surface stress reaching −887 MPa, increasing to −1341 MPa at 61 µm, and then transitioning to tensile stress at a deeper layer of 805 µm. As shown in Figure 11, the residual stress σ_y_ induced by burnishing was greater than that along the burnishing direction on the B-B’ plane (σ_x_). This finding aligns with the observed greater depth of effective grain straining/modification in the direction normal to burnishing, as shown in Figure 10. This beneficial compressive residual stress is expected to improve the fatigue and corrosion resistance of DEDed Stellite 11 alloys.

### 3.5. FWHM Analysis

**Figure 12:**
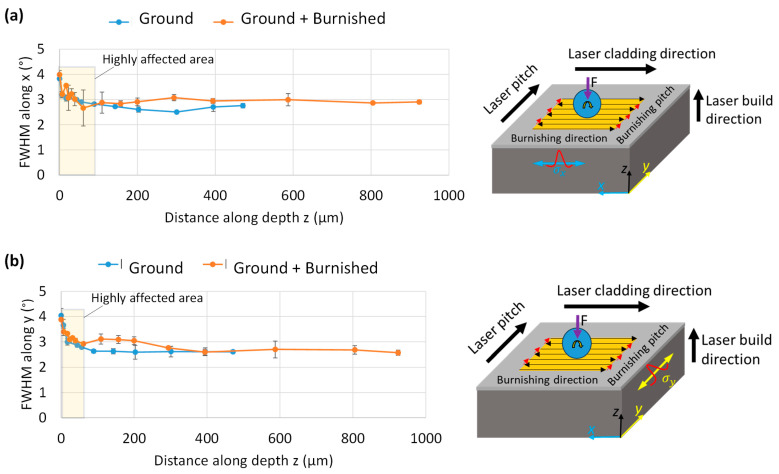
Comparison of FWHM with respect to distance along depth induced by grinding and burnishing along (**a**) the burnishing direction (x) on B-B’ plane and (**b**) normal to burnishing direction (y) on A-A’ plane. Images on right side in (**a**,**b**) show an illustration of burnishing path and XRD FWHM peak measurement directions.

Further, FWHM (Full Width at Half Maximum) serves as an indicator of the degree of grain straining in relation to grain size, with a higher FWHM corresponding to a smaller grain size. As shown in Figure 12a, the trend of FWHM along the *x*-axis (in the burnishing direction) for both the ground and burnished specimens remains consistent up to a depth of 110 µm. At the top surface, the FWHM value (3.9°) is the highest and decreases gradually with increasing depth. Below 110 µm, the burnished surface exhibits a higher FWHM (3.1°) along the *x*-axis (burnishing direction) compared to the ground surface (2.5°).

In contrast, Figure 12b illustrates that the trend for FWHM along the *y*-axis (perpendicular to the burnishing direction) is similar for both the burnished and ground surfaces up to a depth of 60 µm. Beyond this point, the FWHM for the burnished surface becomes higher than that of the ground surface, continuing this trend until a depth of 400 µm, after which the effect of burnishing becomes negligible. This indicates that the burnishing induces more significant and deeper grain straining along the direction normal to the burnishing direction. These results are aligned with XRD results for both the ground and burnished surfaces on B-B’ and A-A’ planes along the burnishing and normal to the burnishing directions, respectively, as presented in Figure 11.

**Figure 13:**
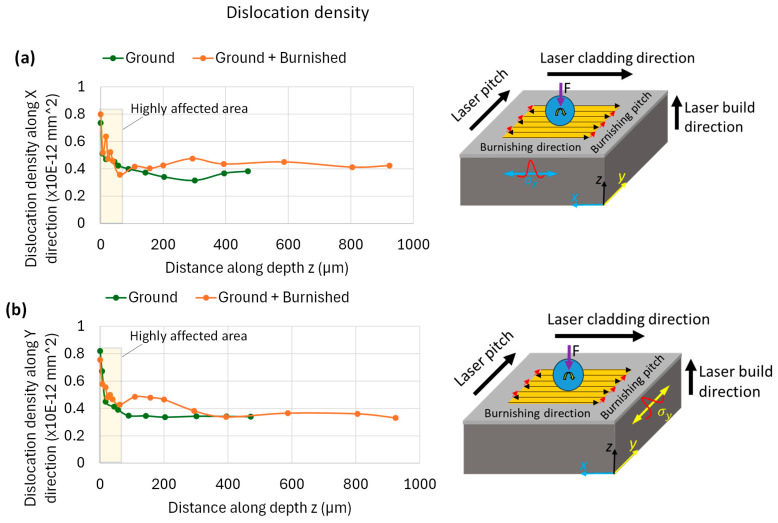
Comparison of the dislocation density with respect to distance along depth induced by grinding and burnishing along (**a**) the burnishing direction (x) on B-B’ plane and (**b**) normal to burnishing direction (y) on A-A’ plane. Images on right side in (**a**,**b**) show an illustration of burnishing path and XRD FWHM peak measurement directions.

Further, based on the FWHM data, the dislocation density along the depth from the tip surface for both ground and burnished specimens was estimated using the Modified Williamson-Hall method [24]. Figure 13 compares the dislocation density along the *x*-axis (along burnishing direction) and along *y*-axis (perpendicular to the burnishing direction). As can be seen from Figure 13, the dislocation density follows the similar trend of FWHM. The burnishing increased the dislocation density over the ground specimen. In particular, the density location density along *y*-axis or along normal to the burnishing direction (on A-A’ plane) is significant and higher beyond 60 µm depth below the top surface, as compared to that along *x*-axis or along the burnishing direction (on B-B’ plane). For instance, at 100 µm depth, the dislocation density along *x*-axis is 0.41 × 10^−12^ mm^2^ while that along *y*-axis is 0.48 × 10^−12^ mm^2^. The dislocation density is associated with hardness, i.e., higher dislocation means higher hardness.

### 3.6. Micro-Hardness Analysis

**Figure 14:**
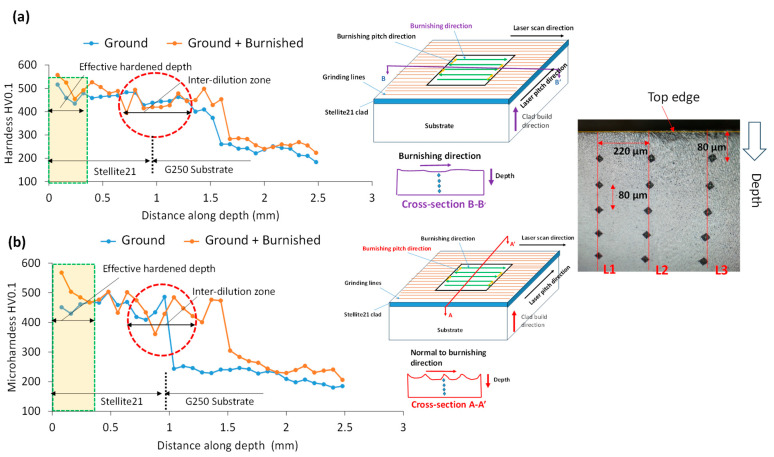
Comparison of microhardness on cross-sectional surfaces cut (**a**) along burnishing direction on B-B’ plane and (**b**) along normal to burnishing on A-A’ plane, as illustrated in schematic diagrams next to the hardness plots. Light yellow boxes in (**a**,**b**) highlight the effective modified depth of hardened material layer due to burnishing while the red circles indicate the sudden hardness increase in heat affected zone near the interface between the substrate and Stellite 21 clad. Right side images indicate the cross-sectional planes on which the hardness was measured on the surface treated specimens.

Figure 14 presents a comparison of microhardness (HV_0.1_) versus depth for cross-sectional surfaces along the burnishing (B-B’ plane) and perpendicular to the burnishing (A-A’ plane) directions. As shown in Figure 14a, burnishing enhanced the maximum hardness along the B-B’ plane by 8%, increasing from 516 on the ground surface to 557 at a depth of up to 320 µm. Beyond this depth, the hardness increase plateaued, aligning with the bulk hardness of Stellite 21.

In contrast, as shown in Figure 14b, the hardness increase along the A-A’ plane (normal to the burnishing direction) was more significant than along the B-B’ plane. Specifically, burnishing increased the hardness by 26%, from 451 on the ground surface to 568, with the depth of modification remaining nearly the same (320 µm) as in the B-B’ plane. This greater hardness increase on the A-A’ plane could be attributed to a higher strain hardening effect in the burnishing direction (along the A-A’ plane). Moreover, these hardness results are consistent to the dislocation density as presented in Figure 13.

Additionally, as shown in Figure 14, sharp hardness peaks, marked by red dashed circles, were observed at a deeper layer of approximately 1 mm below the top surface. In this study, the thickness of the laser-cladded Stellite 21 layer was approximately 1 mm, and the burnishing force had a negligible effect on these hardness peaks. These abrupt increases in hardness likely result from the hardened layer formed within the heat-affected dilution zone at the interface between the substrate and the cladding. This phenomenon is common in DED processes. Below this dilution area, the hardness of the underlying G250 substrate, approximately 230, prevailed.

## 4. Discussion

Surface integrity plays a critical role in the functional performance of metal components. This study demonstrates that the surface integrity of DEDed Co-based Stellite 21 alloys can be significantly enhanced through ball burnishing, making the material more suitable for practical applications. The directional effect of ball burnishing, relative to the grinding or laser scanning direction, on plastic deformation is a key factor in determining an effective post-processing strategy.

Our findings show that the burnishing can substantially smooth the surface by eliminating grinding marks when performed perpendicular to the grinding or laser scanning direction. Additionally, when burnishing is applied perpendicular to the grinding lines, we observe a deeper grain modification, increased compressive stress, and higher hardness along the cross-sectional surface of the material. This can be attributed to the fact that, when burnishing occurs normal to the grinding lines, the burnishing ball pushes the peaks of the ground surface into the valleys via plastic deformation. As the contact area between the ball and the surface peaks is smaller, the contact stress and plastic deformation are higher, resulting in greater grain misorientations and the formation of more equiaxed grains extending from the surface into deeper layers.

**Figure 15:**
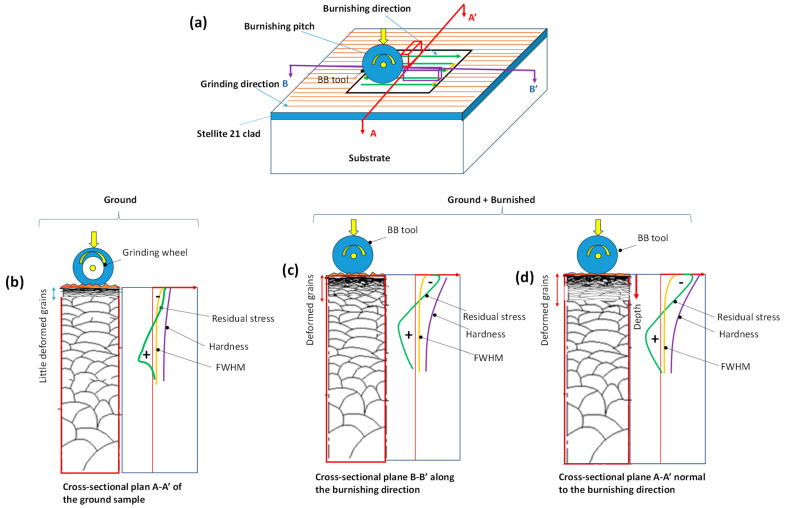
Illustration of plastically induced grain modification on the cross-section planes of the treated samples: (**a**) definition of cross-sectional planes; (**b**) ground sample A-A’ plane; (**c**) ground + burnished sample B-B’ plane (along the burnishing); and (**d**) ground + burnished sample A-A’ plane (normal to the burnishing direction or burnishing pitch direction).

Conversely, when the burnishing ball follows the grinding lines or is aligned with the burnishing direction, the tip of the ball primarily contacts the surface valleys. This larger contact area leads to lower contact pressure and, consequently, less plastic deformation in the material. A comparative analysis of surface and grain modification by grinding and burnishing along cross-sectional planes, both parallel and perpendicular to the burnishing direction, and the resulting surface integrity improvement, is shown in Figure 15.

Regardless of the cross-sectional plane, the burnishing increases compressive residual stress on both the surface and along the depth, due to the micro-straining of the grains. This mechanism of grain modification aligns with the theory proposed by Williamson and Hall, which states that higher micro-strain leads to higher compressive residual stress [24]. Higher micro-strain is also correlated with an increased full width at half maximum (FWHM) along the direction perpendicular to the burnishing process. Our results are consistent with those of [25], who reported that slide and rotational burnishing lead to higher micro-strain and subsequently increased compressive stress.

A noticeable increase in microhardness at the surface and along the cross-section was observed due to the work-hardening effect induced by plastic strain from both grinding and burnishing. The burnishing effect on hardness improvement is most pronounced on the cross-sectional surface of plane A-A’, which is normal to the burnishing direction. Although no grain refinement within the dendritic microstructures was observed after burnishing at the applied force, the grains exhibited geometric modifications and a high density of dislocations due to plastic deformation. This likely explains the observed hardness improvement at the top surface, followed by a gradual decay in hardness.

Furthermore, the higher hardness on the A-A’ plane compared to the B-B’ plane is likely attributed to the intrinsic nature of each track deposition and the overlap between tracks. This leads to slight differences in microstructure and hardness along the laser pitch or burnishing direction. Therefore, it can be concluded that hardness anisotropy may arise to some extent after ball burnishing of the laser-cladded Stellite 21.

The hardness profiles along the depth from the top surface closely align with the grain-straining Full Width at Half Maximum (FWHM) and the dislocation density results presented in Figure 12 and Figure 13, respectively. This correlation between hardness and grain modification due to roller burnishing, or similar plastic surface modification processes, e.g., ultrasonic surface rolling on Stellite 6 has been documented in the literature [22]. Similar to rolling, Flores-Garcia et al. [26] applied the laser shock peening (LSP) on Stellite 6 alloys and shown an increase of the compressive stress from 75 MPa to 250 MPa with a modification layer up to 250 µm from the top surface. However, the effect of LSP is lower than the effect of ball burnishing presented in our work–higher and deep compressive stress (−1341 MPa and 805 µm) along the plane normal to the burnishing direction, as shown in Figure 11.

While grinding induced some compressive stress from the top layer down to deep into subsurface, the degree of grain modification was relatively marginal. This is because the grinding force is small, and therefore insufficient to cause significant changes in grain orientation at deeper layer. The ground specimens exhibited uniformly distributed, elongated grains. Although the grinding heat softens the material, its rapid cooling can generate residual tensile stress, which may contribute to the initiation and propagation of fatigue failure. This phenomenon regarding grain evolution due to grinding in Directed Energy Deposited (DED) 718 alloys was further explored and confirmed in a study by [25].

## 5. Conclusions

This paper investigates the impact of burnishing on the surface integrity and residual stress of DEDed Stellite 21 alloys, with a particular emphasis on changes in surface properties along two directional planes relative to the burnishing direction. The key findings are summarized as follows:

- The burnishing further improved the surface finish of the ground specimen by plastically deforming the surface peaks and valleys.
- The burnishing modified the Co-based dendrite structure beneath the surface, creating a flattened cellular/columnar grain structure, but the modification was more pronounced on the plane perpendicular to the burnishing direction, compared to the plane parallel to the burnishing direction.
- The burnishing effectively induced higher and deeper compressive stresses. The maximum in-depth compressive stress was higher on the plane normal to the burnishing direction than on the plane aligned with the burnishing direction, as was supported by the FWHM results.
- Due to grain refinement and potential dislocation movement, the hardness improvement was greater in the cross-sectional plane normal to the burnishing direction than along the burnishing direction.

Finally, it is imperative to say that the burnishing has a directional effect on material deformation, which in turn results in variations in the surface integrity of DEDed Stellite 21 alloys. This is primarily influenced by the contact pressure and the interaction between the burnishing tool and the surface peaks and valleys of the specimen, relative to the grinding/machining lines of the specimen. The degree of deformation and surface integrity depends on the initial surface roughness/texture and the burnishing processing parameters. This needs to be more considered for postprocessing of high strength and hard materials such as Stellite or Inconel alloys. The findings from this study provide valuable insights for designing burnishing path strategies, enabling the post-processing of DEDed Stellite 21 alloys with tailored surface integrity and functional performance for real-world applications. One possibility is the functional performance properties of the components, e.g., wear, friction, corrosion, impact resistance should be targeted to the modified surface plane normal to the burnishing direction.

## Figures and Tables

**Table 1:** Laser direct energy deposition parameters used in this study.

Laser Spot	Laser Power	Laser Scanning Speed	Sheilding Gas Flowrate	Carrier Gas Flow Rate	Nozzle Stand-Off Distance	Pitching Disance
4.8 mm	5 kW	1.5 m/min	Helium and Argon (5 LPM)	Nitrogen (7 LPM)	17–19 mm	2 mm

**Table 2:** Chemical composition (wt%) of DEDed Stellite 21 and substrate G250.

Material	Fe	Cr	Co	Ni	Mo	Mn	C	Si	P	S	Al
DEDed Stellite 21 alloy	<2.0	28	Bal.	3	5	-	<1	-	-	-	-
Substrate G250	Bal.	-	-	-	-	0.25	0.07	0.03	0.02	0.02	0.07

**Table 3:** Residual stress measurement parameters.

Parameter	Value
Diffraction condition	Mn Kα X-ray tube 18 kV and 40 mA
X-ray beam size	2 mm
X-ray wavelength	2.103 Angstrom
X-ray penetration	up to 5 µm
Bragg’s angle 2-theta	150.41°
Plane {hkl}	{211}
Bragg’s d-spacing	1.0876296 Angstrom
-S1(v/E)	0.98 × 10^−6^ [1/[MPa]]
S2/2(1 + v)/E	5.63 × 10^−6^ [1/[MPa]]
Acquisition	Ψ mode, 7 Ψ angles from −30 to +30°, exposure of 8 s

## Data Availability

The original contributions presented in this study are included in the article. Further inquiries can be directed to the corresponding author.
